# Fuzzy Classification of High Resolution Remote Sensing Scenes Using Visual Attention Features

**DOI:** 10.1155/2017/9858531

**Published:** 2017-07-06

**Authors:** Linyi Li, Tingbao Xu, Yun Chen

**Affiliations:** ^1^School of Remote Sensing and Information Engineering, Wuhan University, Wuhan 430079, China; ^2^Fenner School of Environment and Society, The Australian National University, Canberra, ACT 2601, Australia; ^3^Commonwealth Scientific and Industrial Research Organization (CSIRO) Land and Water, Canberra, ACT 2601, Australia

## Abstract

In recent years the spatial resolutions of remote sensing images have been improved greatly. However, a higher spatial resolution image does not always lead to a better result of automatic scene classification. Visual attention is an important characteristic of the human visual system, which can effectively help to classify remote sensing scenes. In this study, a novel visual attention feature extraction algorithm was proposed, which extracted visual attention features through a multiscale process. And a fuzzy classification method using visual attention features (FC-VAF) was developed to perform high resolution remote sensing scene classification. FC-VAF was evaluated by using remote sensing scenes from widely used high resolution remote sensing images, including IKONOS, QuickBird, and ZY-3 images. FC-VAF achieved more accurate classification results than the others according to the quantitative accuracy evaluation indices. We also discussed the role and impacts of different decomposition levels and different wavelets on the classification accuracy. FC-VAF improves the accuracy of high resolution scene classification and therefore advances the research of digital image analysis and the applications of high resolution remote sensing images.

## 1. Introduction

With the rapid development of satellite and sensor technologies, remote sensing has become an important and efficient means to collect earth spatial information in recent years [1–4]. Remote sensing images with high spatial resolutions can be acquired from satellites, such as IKONOS, QuickBird, and WorldView [5, 6]. High resolution remote sensing images provide us with a great deal of information on texture structures and spatial details. The improvement in spatial resolution also increases the intraclass variability of land-cover classes and reduces the interclass variability between different classes [7], which increase the fuzziness of classification and pose a big challenge for automatic classification of remote sensing scenes. Remote sensing scenes are the separated subareas extracted from remote sensing images and possess specific semantic meanings, such as farmlands and residential areas. Remote sensing scene classification is a process to classify specific scenes in remote sensing images, which is essential to many remote sensing applications and has attracted much attention in recent years [8–10]. Various classification methods have been developed, which can be applied to remote sensing scene classification, such as minimum distance method [11], maximum likelihood method [11], neural network methods [12–16], fuzzy methods [17–21], support vector machine methods [21–23], particle swarm optimization methods [19, 24], artificial immune methods [25, 26], and Markov model methods [27–29]. However, due to the complex texture structures and spatial details in high resolution remote sensing scenes, scene classification is still a difficult task. Remote sensing scene classification methods based on visual attention may provide potential solutions to resolve this issue.

Visual attention is an important characteristic of the human visual system [30]. The human visual system can be easily attracted by salient details of an image and recognize objects or scenes in the image. Visual saliency measures to what extent details in an image attract human attention [31]. In the past twenty years, visual attention has become one of the hot spots in the relevant research and applications of artificial intelligence [32–36]. In 1998, Itti et al. proposed a visual attention model [32], which was based on the attention mechanism of the human visual system. The Itti visual attention model can be used to extract a variety of features from input images, such as brightness and color. Then these features were analyzed and consolidated to generate saliency maps. Walther and Koch further developed the saliency model proposed by Itti et al. They introduced a feedback mechanism in generating saliency maps for object recognition [33]. Achantay et al. proposed a frequency-tuned method to compute pixel saliency directly and detect salient regions [34]. Hou and Zhang designed a fast method to detect image saliency by exploring spectral components in an image [35]. Tian et al. proposed a color saliency model to detect salient objects in natural scenes [36]. In their color saliency model, different color features were extracted and analyzed. For different color features, two efficient saliency measurements were employed to compute different saliency maps. And a feature combination strategy was presented to combine multiple saliency maps into one integrated saliency map. Scene feature extraction is a key step in scene classification, which affects the classification accuracy. When the human visual system observes and classifies scenes, it is usually through a multiscale process. However, attempts to extract visual attention features through a multiscale process for scene classification are relatively rare in literatures.

The assumption of this study is that visual attention features could be extracted through a multiscale process for high resolution remote sensing scene classification. Fuzzy theory is an effective mathematical tool to process fuzzy and complex information [17–21], which could be suitable for high resolution remote sensing scene classification. Therefore, the fuzzy classification method [17–19] is preferred in this study. The main goals of this study are (1) to propose a novel visual attention feature extraction algorithm based on wavelet transform, which extracts visual attention features through a multiscale process; (2) to apply a fuzzy classification method (FC) using visual attention features (VAF) to achieve an improved accuracy in the scene classification; (3) to compare and evaluate the effects of FC-VAF with four traditional classification methods using IKONOS, QuickBird, and ZY-3 remote sensing scenes; and (4) to discuss the parameter sensitivity of FC-VAF.

## 2. Methodology

### 2.1. Wavelet Transform-Based Visual Attention Feature Extraction

#### 2.1.1. Basic Principle of Wavelet Transform

**Figure 1:**
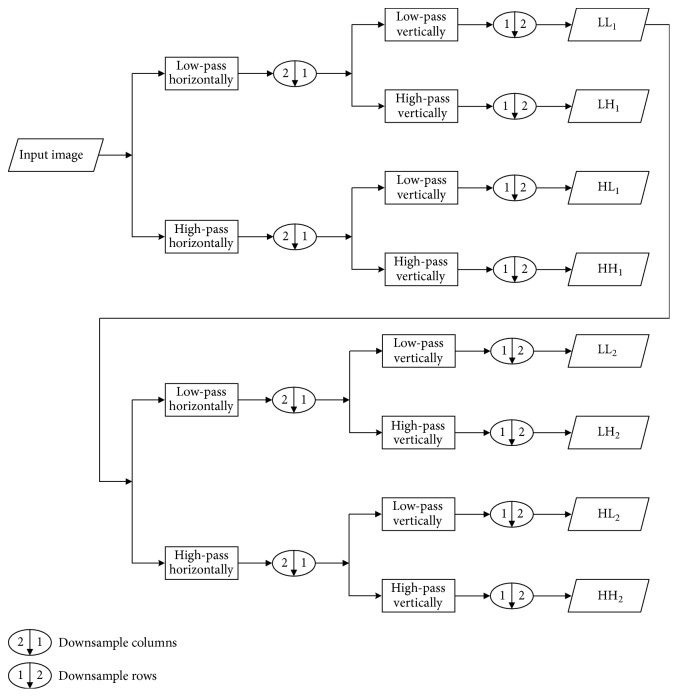
Two-level two-dimensional discrete wavelet decomposition of an image.

The wavelet analysis is a powerful mathematical tool to obtain decomposition, reconstruction, and a multiscale representation of signals [37–39]. It introduces inherent scaling and good identification of signals, which is relevant to the human perception. A digital image is regarded as a two-dimensional discrete signal and can be decomposed and reconstructed by the two-dimensional discrete wavelet transform. The two-dimensional discrete wavelet transform allows good localization in both the frequency and spatial domain. The image can be decomposed into multiple levels using wavelet basis functions. It can be considered as a chain of successive levels of decomposition of the image by applying the one-dimensional discrete wavelet transform in the horizontal and vertical directions [37–39]. Two-level two-dimensional discrete wavelet decomposition of an image is illustrated in Figure 1. There are several popular wavelets in the field of the wavelet analysis, such as Daubechies wavelets, Symlets wavelets, and Discrete Meyer wavelet [39]. Different wavelets lead to different wavelet decomposition effects and application results.

#### 2.1.2. Visual Attention Feature Extraction through a Multiscale Process

Wavelet transform can obtain the multiscale representation of images. Therefore, a novel visual attention feature extraction algorithm based on wavelet transform is proposed, which extracts visual attention features from the saliency maps of remote sensing scenes through a multiscale process.

Visual saliency in an image measures to what extent details attract human attention [31]. Tian et al. proposed a color saliency model to detect salient objects in natural scenes [36]. In their color saliency model, different color features were extracted and analyzed. For different color features, two efficient saliency measurements were proposed to compute different saliency maps. And a feature combination strategy was presented to combine multiple saliency maps into one integrated saliency map. We adopt the color saliency model above to obtain the integrated saliency map *s*(*x*, *y*) for an image *f*(*x*, *y*) as follows [36]:

(1) sx,y=∑m=1Mwm∗smx,y∑m=1Mwm=1smx,y=11+exp⁡−dmx,y/dm¯dmx,y=fmx,y−fm¯,

where *M* = 3; *s*_*m*_(*x*, *y*)  (*m* = 1,2, 3) represent the saliency maps of the intensity, hue, and saturation components of the image, respectively; *w*_*m*_  (*m* = 1,2, 3) represent the weight values of *s*_*m*_(*x*, *y*)  (*m* = 1,2, 3), respectively; *f*_*m*_(*x*, *y*)  (*m* = 1,2, 3) represent the intensity, hue, and saturation components of the image, respectively; $\bar{f_{m}}$ is the average value of *f*_*m*_(*x*, *y*); $\bar{d_{m}}$ is the average value of *d*_*m*_(*x*, *y*).

The visual attention features are extracted from an integrated saliency map as follows.

**Figure 2:**
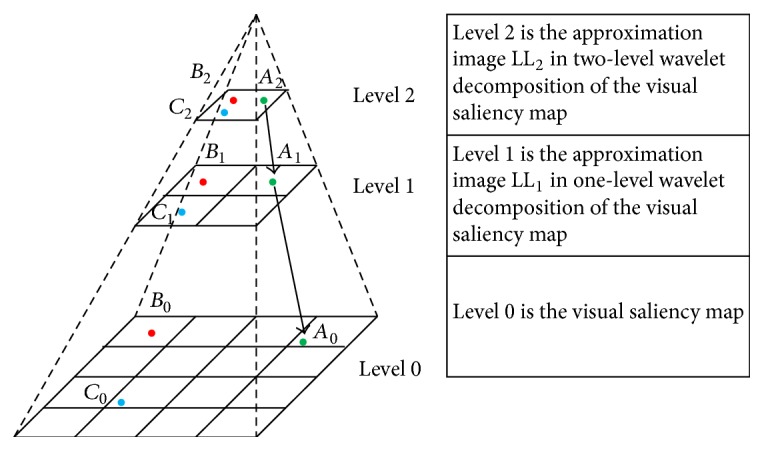
The multiscale representation of an integrated saliency map for visual attention feature extraction (*N* = 2).

(a) The integrated saliency map is decomposed by* N*-level two-dimensional discrete wavelet transform. The multiscale representation of the integrated saliency map is obtained and composed by LL_1_, LL_2_,…, LL_*N*_. The multiscale representation of an integrated saliency map for visual attention feature extraction is illustrated in Figure 2, where *N* = 2.

(b) Visual attention focuses are extracted in the top level of the multiscale representation. The salient points in the top level are extracted based on the saliency values of the points. The human visual system can be easily attracted by the most salient point. Therefore, the most salient point is selected as the first and current visual attention focus. Then visual attention is shifted among the salient points in the top level. The next visual attention focus is the unselected salient point which is closest to the current visual attention focus. For example, there are three salient points in Figure 2. The most salient point *A*_2_ is selected as the first and current visual attention focus. Then select the salient point *B*_2_ as the second visual attention focus because it is closer to *A*_2_ than *C*_2_.

(c) Visual attention is shifted from the top level to the low level of the multiscale representation. Take the visual attention focus *A*_2_ in Figure 2, for example. According to the position relation between two adjacent levels of the multiscale representation, *A*_2_ in LL_2_ corresponds to a small region in LL_1_. Select the point with maximal value in the region as the corresponding visual attention focus *A*_1_. In the same way, we can obtain the visual attention focus *A*_0_ in the visual saliency map.

(d) The saliency values of the visual attention focuses in the visual saliency map are used as the visual attention features for scene classification. In Figure 2, the saliency values of *A*_0_, *B*_0_, and *C*_0_ are used as the visual attention features.

### 2.2. Fuzzy Classification of Remote Sensing Scenes

We apply the fuzzy classification method [17–19] using visual attention features to achieve an improved accuracy of scene classification. The classification procedure is described as follows.

(a) Multiple original features are extracted from the samples of remote sensing scenes, including gray level cooccurrence matrix features [40], Laws texture energy features [40], and visual attention features. These features consist of feature vectors, which represent the corresponding scenes in the recognition process.

(b) The features are transformed into fuzzy features using the standard S-function as follows:

(2) $$\begin{array}{c} \mu_{Y}\left(\;y\;\right)=\left\{\begin{array}{cc} 0 & y<a\\ 2\times {\left[\frac{\left(y-a\right)}{\left(c-a\right)}\right]}^{2} & a\leq y<b\\ 1-2\times {\left[\frac{\left(c-y\right)}{\left(c-a\right)}\right]}^{2} & b\leq y<c\\ 1 & y\geq c, \end{array}\right. \end{array}$$

where *a*, *b*, and *c* are the fuzzy parameters; *b* = (*a* + *c*)/2.

(c) Fuzzy class centers are obtained by using the mean value method. Suppose *c*_*ij*_ is the *j*th component of the class center of the *i*th class, *N* is the number of the training samples of the *i*th class, and *t*_*nj*_ is the *j*th component of the feature vector of the training sample *n*; then *c*_*ij*_ is computed as follows:

(3) $$\begin{array}{c} c_{ij}=\frac{1}{N}\overset{N}{\underset{n=1}{\sum }}t_{nj}, \end{array}$$

where *j* = 1,2,…, *M*; *M* is the dimension of the feature vectors of the samples.

(d) Test samples are classified using Euclidean fuzzy closeness degree on the basis of the fuzzy closeness principle [18].

(e) Fuzzy classification results are assessed using overall accuracy (OA), Kappa coefficient (KC), average producer's accuracy (APA), and average user's accuracy (AUA) based on confusion matrices [41, 42].

**Figure 3:**
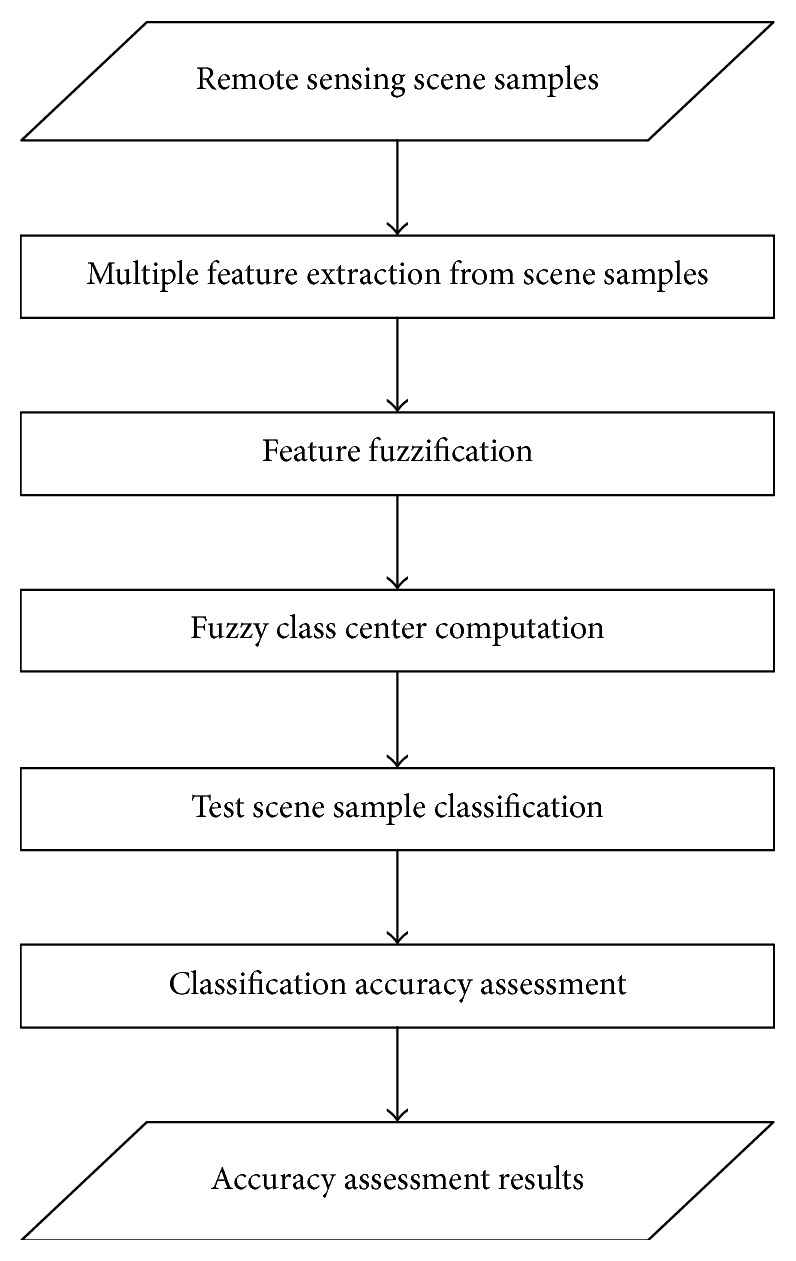
Flowchart of the fuzzy classification process.

A flowchart of the fuzzy classification process is shown in Figure 3.

## 3. Case Study

### 3.1. Materials

**Figure 4:**
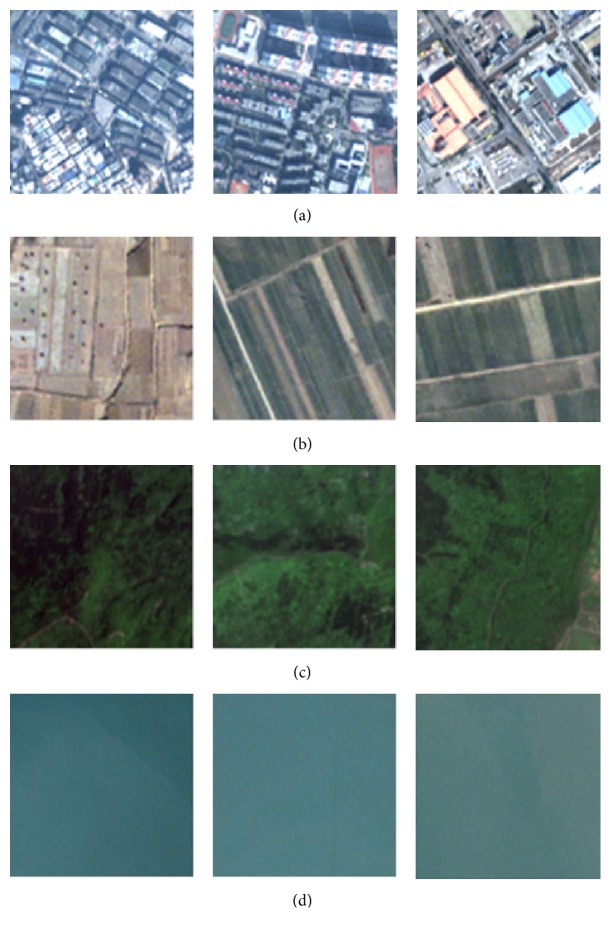
Representative samples of remote sensing scenes. (a) Residential areas; (b) farmlands; (c) woodlands; (d) water areas.

In order to validate the effectiveness of FC-VAF, 80 samples of remote sensing scenes were selected as the experimental data from widely used high spatial resolution remote sensing images, including IKONOS, QuickBird, and ZY-3 images. The samples consist of four classes, which are residential areas, farmlands, woodlands, and water areas, respectively. Each class has 20 samples where 10 samples are used as the training samples and all are used as the test samples. The size of the samples is 100 × 100 pixels. Representative samples of remote sensing scenes are shown in Figure 4.

### 3.2. Methods and Results

To demonstrate the effectiveness of FC-VAF, comparisons were carried out between FC-VAF and scene classification based on four traditional algorithms. The four methods for comparison are standard backpropagation neural network classification (SBPC), adaptive learning rule backpropagation neural network classification (ALRBPC), general regression neural network classification (GRNNC), and fuzzy classification (FC). Four gray level cooccurrence matrix features and four Laws texture energy features were extracted from these samples for all scene classification methods. The Euclidian closeness degree measurement was adopted in both FC and FC-VAF. Symlets wavelet was adopted in FC-VAF. The main parameters of different methods are shown in Table 1.

We compared the results of different scene classification methods using the measures of OA, KC, APA, and AUA.  Table 2 shows the performances in terms of the classification accuracy derived by SBPC, ALRBPC, GRNNC, FC, and FC-VAF. From Table 2, we can see that GRNNC outperformed FC and ALRBPC using OA, KC, APA, and AUA, while SBPC was the worst performer. FC-VAF obtained the best classification results among the five methods according to the values of OA, KC, APA, and AUA. For example, the OA values of SBPC, ALRBPC, GRNNC, FC, and FC-VAF are 76.3%, 78.8%, 82.5%, 80.0%, and 85.0%, respectively. The KC values of SBPC, ALRBPC, GRNNC, FC, and FC-VAF are 0.683, 0.717, 0.767, 0.733, and 0.800, respectively. FC-VAF can obtain satisfactory classification results in such images, because FC-VAF is on the basis of fuzzy theory and utilizes visual attention features in the process of classification.

## 4. Discussion

### 4.1. Discussion of the Effects of Wavelet Decomposition Levels

**Figure 5:**
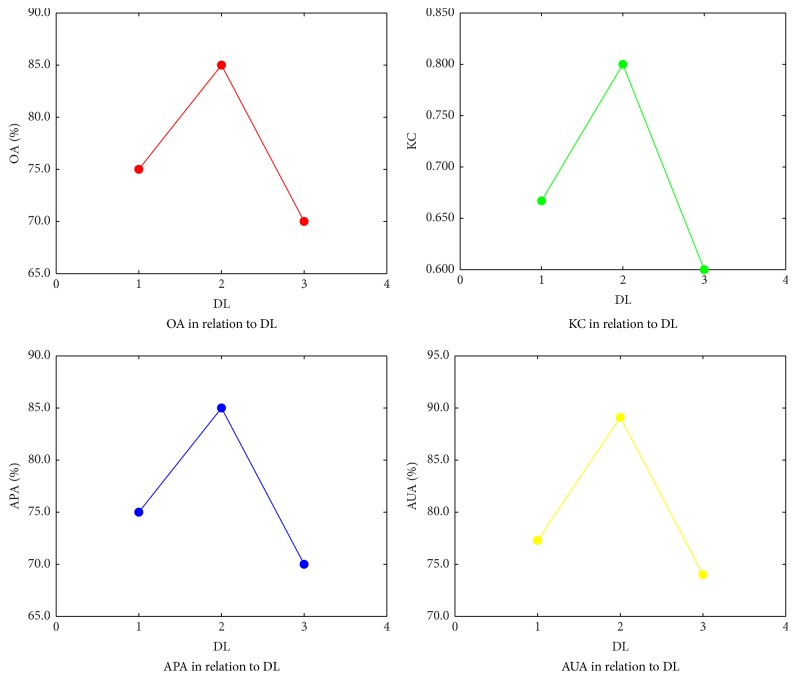
The effects of different wavelet decomposition levels (DL) on the classification accuracy. OA represents overall accuracy, KC represents Kappa coefficient, APA represents average producer's accuracy, and AUA represents average user's accuracy.

The decomposition level (DL) of wavelets is the key parameter of FC-VAF, which affects the accuracy of scene classification. The scene classification accuracy of FC-VAF related to DL was analyzed and discussed. The 80 samples of scenes in the case study were used with different DL values (DL = 1,2, 3). Other parameters of FC-VAF were kept the same as those in the case study. The classification accuracy of FC-VAF for each DL value is shown in Figure 5. It shows that, with the increase of the DL value, the OA value increases to the maximum 85.0% when DL is 2 and then decreases. KC, APA, and AUA have similar trends as that of OA. Therefore, the optimal value of DL is 2 among the three values for FC-VAF in this application.

### 4.2. Discussion of the Effects of Different Wavelets

**Figure 6:**
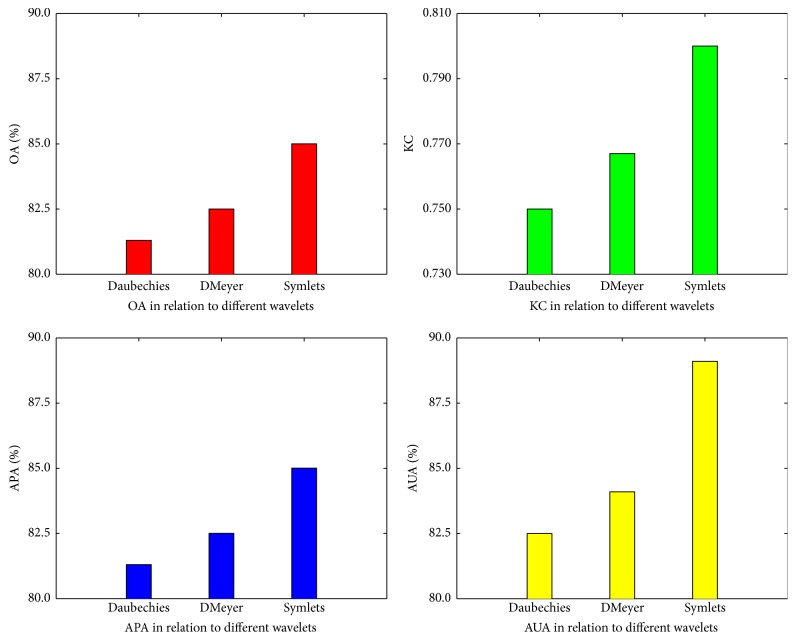
The effects of different wavelets on the classification accuracy. OA represents overall accuracy, KC represents Kappa coefficient, APA represents average producer's accuracy, and AUA represents average user's accuracy.

Different wavelets lead to different wavelet decomposition effects, which affect the classification accuracy of FC-VAF. The scene classification accuracy of FC-VAF related to wavelets was analyzed and discussed. The 80 samples of scenes in the case study were used with different wavelets. Other parameters of FC-VAF were kept the same as those in the case study. The classification accuracy of FC-VAF using different wavelets is shown in Figure 6. It shows that DMeyer wavelet outperformed Daubechies wavelet using the measures of OA, KC, APA, and AUA, while Symlets wavelet was the best performer. For example, the OA values of Daubechies wavelet, DMeyer wavelet, and Symlets wavelet are 81.3%, 82.5%, and 85.0%, respectively. Therefore, Symlets wavelet is optimal among the three wavelets for FC-VAF in this application.

## 5. Conclusions

In this study, a novel visual attention feature extraction algorithm was proposed, which extracted visual attention features through a multiscale process. And a fuzzy classification method using visual attention features (FC-VAF) was developed to perform high resolution remote sensing scene classification. FC-VAF was evaluated by using 80 samples of remote sensing scenes, which were selected from widely used high resolution remote sensing images, including IKONOS, QuickBird, and ZY-3 images. FC-VAF achieved more accurate classification results than four traditional classification methods according to the measures of OA, KC, APA, and AUA. The OA values of SBPC, ALRBPC, GRNNC, FC, and FC-VAF are 76.3%, 78.8%, 82.5%, 80.0%, and 85.0%, respectively. The KC values of SBPC, ALRBPC, GRNNC, FC, and FC-VAF are 0.683, 0.717, 0.767, 0.733, and 0.800, respectively. The classification accuracy of FC-VAF related to the decomposition level and to the wavelets was discussed.

FC-VAF can extract visual attention features through a multiscale process and improve the accuracy of scene classification in high resolution remote sensing images. Therefore, FC-VAF not only advances the research of visual attention models and digital image analysis methods, but also promotes the applications of high resolution remote sensing images. Possible further development of the study will focus on the integration of FC-VAF and other intelligent algorithms to further improve the accuracy of high resolution remote sensing scene classification.

## Figures and Tables

**Table 1:** Main parameters of different methods.

Method	Parameter description	Parameter value
SBPC	Number of hidden layers	1
	Number of neurons in hidden layers	15
	Learning rate	0.01
	Maximum number of epochs to train	5000

ALRBPC	Number of hidden layers	1
	Number of neurons in hidden layers	15
	Learning rate	0.01
	Ratio to increase learning rate	1.05
	Ratio to decrease learning rate	0.7
	Maximum number of epochs to train	1000

GRNNC	Spread parameter	0.5

FC	Fuzzy parameter *a*	0.2
	Fuzzy parameter *c*	0.8

FC-VAF	Fuzzy parameter *a*	0.2
	Fuzzy parameter *c*	0.8
	Level of wavelet decomposition	2
	Number of VAF	4

**Table 2:** Comparisons of different scene classification methods.

Methods	Scene classification accuracy indicators			
	Overall accuracy (OA) (%)	Kappa coefficient (KC)	Average producer's accuracy (APA) (%)	Average user's accuracy (AUA) (%)
SBPC	76.3	0.683	76.3	78.4
ALRBPC	78.8	0.717	78.8	81.5
GRNNC	82.5	0.767	82.5	86.8
FC	80.0	0.733	80.0	82.4
FC-VAF	85.0	0.800	85.0	89.1

## References

[1] Li L., Chen Y., Xu T., Liu R., Shi K., Huang C. (2015). Super-resolution mapping of wetland inundation from remote sensing imagery based on integration of back-propagation neural network and genetic algorithm. *Remote Sensing of Environment*.

[2] Chen Y., Wang B., Pollino C. A., Cuddy S. M., Merrin L. E., Huang C. (2014). Estimate of flood inundation and retention on wetlands using remote sensing and GIS. *Ecohydrology*.

[3] Li L., Chen Y., Yu X., Liu R., Huang C. (2015). Sub-pixel flood inundation mapping from multispectral remotely sensed images based on discrete particle swarm optimization. *ISPRS Journal of Photogrammetry and Remote Sensing*.

[4] Liu R., Chen Y., Wu J. (2016). Assessing spatial likelihood of flooding hazard using naïve Bayes and GIS: a case study in Bowen Basin, Australia. *Stochastic Environmental Research and Risk Assessment*.

[5] Jeong J., Yang C., Kim T. (2015). Geo-positioning accuracy using multiple-satellite images: IKONOS, QuickBird, and KOMPSAT-2 stereo images. *Remote Sensing*.

[6] Tarantino C., Adamo M., Lucas R., Blonda P. (2016). Detection of changes in semi-natural grasslands by cross correlation analysis with WorldView-2 images and new Landsat 8 data. *Remote Sensing of Environment*.

[7] Demir B., Bruzzone L. (2016). Histogram-Based Attribute Profiles for Classification of Very High Resolution Remote Sensing Images. *IEEE Transactions on Geoscience and Remote Sensing*.

[8] Zhao B., Zhong Y., Xia G.-S., Zhang L. (2016). Dirichlet-derived multiple topic scene classification model for high spatial resolution remote sensing imagery. *IEEE Transactions on Geoscience and Remote Sensing*.

[9] Zhong Y., Zhu Q., Zhang L. (2015). Scene classification based on the multifeature fusion probabilistic topic model for high spatial resolution remote sensing imagery. *IEEE Transactions on Geoscience and Remote Sensing*.

[10] Ghosh D., Kaabouch N. (2014). A survey on remote sensing scene classification algorithms. *WSEAS Transactions on Signal Processing*.

[11] ERDAS IMAGINE http://www.hexagongeospatial.com/support/documentation.

[12] Ressel R., Frost A., Lehner S. (2015). A Neural Network-Based Classification for Sea Ice Types on X-Band SAR Images. *IEEE Journal of Selected Topics in Applied Earth Observations and Remote Sensing*.

[13] Li L., Xu T., Chen Y. (2016). Improved urban flooding mapping from remote sensing images using generalized regression neural network-based super-resolution algorithm. *Remote Sensing*.

[14] Taravat A., Del Frate F., Cornaro C., Vergari S. (2015). Neural networks and support vector machine algorithms for automatic cloud classification of whole-sky ground-based images. *IEEE Geoscience and Remote Sensing Letters*.

[15] Vasuki P., Roomi S. M. M. (2013). Automatic target classification of man-made objects in synthetic aperture radar images using Gabor wavelet and neural network. *Journal of Applied Remote Sensing*.

[16] Goltsev A., Gritsenko V. (2012). Investigation of efficient features for image recognition by neural networks. *Neural Networks*.

[17] Yang A.-M., Li X.-G., Zhou Y.-M., Hu Y.-F. (2008). Fuzzy classifier based on support vector machine. *Journal of System Simulation*.

[18] Liu S., Hou H., Zhang H. (2004). Research of pattern recognition classification based on fuzzy theory for stored producted insects. *Computer Engineering and Applications*.

[19] Li L., Li D. Fuzzy classification of remote sensing images based on particle swarm optimization.

[20] Si X., Peng Z., Yuan H., Chen G. (2012). Research on cucumber downy mildew images classification based on fuzzy pattern recognition. *Sensor Letters*.

[21] Jenicka S., Suruliandi A. (2014). Fuzzy texture model and support vector machine hybridization for land cover classification of remotely sensed images. *Journal of Applied Remote Sensing*.

[22] Liu H., Li S. (2013). Decision fusion of sparse representation and support vector machine for SAR image target recognition. *Neurocomputing*.

[23] Li L., Chen Y., Gao H., Li D. (2012). Automatic recognition of village in remote sensing images by support vector machine using co-occurrence matrices. *Sensor Letters*.

[24] Ding X., Qiu H. (2015). Recognition model of oil reservoir based on chaotic particle swarm optimization. *International Journal of Earth Sciences and Engineering*.

[25] Zhong Y., Zhang L. (2011). An Adaptive Artificial Immune Network for Supervised Classification of Multi-/Hyperspectral Remote Sensing Imagery. *IEEE Transactions on Geoscience and Remote Sensing*.

[26] Dudek G. (2012). An artificial immune system for classification with local feature selection. *IEEE Transactions on Evolutionary Computation*.

[27] Yuan Y., Meng Y., Lin L. (2015). Continuous change detection and classification using hidden Markov model: A case study for monitoring urban encroachment onto farmland in Beijing. *Remote Sensing*.

[28] Qiao Y., Weng L. (2015). Hidden markov model based dynamic texture classification. *IEEE Signal Processing Letters*.

[29] Costa G. A. O. P., Feitosa R. Q. (2014). A generalized fuzzy Markov chain-based model for classification of remote-sensing multitemporal images. *International Journal of Remote Sensing*.

[30] Li L., Ren J., Wang X. (2015). Fast cat-eye effect target recognition based on saliency extraction. *Optics Communications*.

[31] Ren Z., Gao S., Chia L.-T., Tsang I. (2014). Region-based saliency detection and its application in object recognition. *IEEE Transactions on Circuits and Systems for Video Technology*.

[32] Itti L., Koch C., Niebur E. (1998). A model of saliency-based visual attention for rapid scene analysis. *IEEE Transactions on Pattern Analysis and Machine Intelligence*.

[33] Walther D. B., Koch C. (2007). Attention in hierarchical models of object recognition. *Progress in Brain Research*.

[34] Achantay R., Hemamiz S., Estraday F., Süsstrunky S. Frequency-tuned salient region detection.

[35] Hou X. D., Zhang L. Q. Saliency detection: a spectral residual approach.

[36] Tian M., Wan S., Yue L. (2009). A color saliency model for salient objects detection in natural scenes. *Lecture Notes in Computer Science (including subseries Lecture Notes in Artificial Intelligence and Lecture Notes in Bioinformatics)*.

[37] Mallat S. G. (1989). Theory for multiresolution signal decomposition: the wavelet representation. *IEEE Transactions on Pattern Analysis and Machine Intelligence*.

[38] Mallat S. G. (1989). Multifrequency Channel Decompositions of Images and Wavelet Models. *IEEE Transactions on Acoustics, Speech, and Signal Processing*.

[39] The MathWorks Inc., Wavelet Toolbox Documentation, 2016, http://cn.mathworks.com/help/wavelet/index.html

[40] Jia Y. (2015). *Digital Image Processing*.

[41] Feng J., Jiao L., Liu F., Sun T., Zhang X. (2016). Unsupervised feature selection based on maximum information and minimum redundancy for hyperspectral images. *Pattern Recognition*.

[42] Garcia-Llamas P., Calvo L., Álvarez-Martínez J. M., Suárez-Seoane S. (2016). Using remote sensing products to classify landscape. A multi-spatial resolution approach. *International Journal of Applied Earth Observation and Geoinformation*.

